# Optimal *In Silico* Target Gene Deletion through Nonlinear Programming for Genetic Engineering

**DOI:** 10.1371/journal.pone.0009331

**Published:** 2010-02-24

**Authors:** Chung-Chien Hong, Mingzhou Song

**Affiliations:** Department of Computer Science, New Mexico State University, Las Cruces, New Mexico, United States of America; Fondazione Telethon, Italy

## Abstract

**Background:**

Optimal selection of multiple regulatory genes, known as targets, for deletion to enhance or suppress the activities of downstream genes or metabolites is an important problem in genetic engineering. Such problems become more feasible to address *in silico* due to the availability of more realistic dynamical system models of gene regulatory and metabolic networks. The goal of the computational problem is to search for a subset of genes to knock out so that the activity of a downstream gene or a metabolite is optimized.

**Methodology/Principal Findings:**

Based on discrete dynamical system modeling of gene regulatory networks, an integer programming problem is formulated for the optimal *in silico* target gene deletion problem. In the first result, the integer programming problem is proved to be *NP*-hard and equivalent to a nonlinear programming problem. In the second result, a heuristic algorithm, called GKONP, is designed to approximate the optimal solution, involving an approach to prune insignificant terms in the objective function, and the parallel differential evolution algorithm. In the third result, the effectiveness of the GKONP algorithm is demonstrated by applying it to a discrete dynamical system model of the yeast pheromone pathways. The empirical accuracy and time efficiency are assessed in comparison to an optimal, but exhaustive search strategy.

**Significance:**

Although the *in silico* target gene deletion problem has enormous potential applications in genetic engineering, one must overcome the computational challenge due to its *NP*-hardness. The presented solution, which has been demonstrated to approximate the optimal solution in a practical amount of time, is among the few that address the computational challenge. In the experiment on the yeast pheromone pathways, the identified best subset of genes for deletion showed advantage over genes that were selected empirically. Once validated *in vivo*, the optimal target genes are expected to achieve higher genetic engineering effectiveness than a trial-and-error procedure.

## Introduction

Selecting *in silico*, in a dynamic model of gene regulatory and metabolic networks, the right target genes for deletion so as to modify phenotypes can substantially expedite and lower the cost of genetic engineering. The target genes for deletion typically play key regulatory roles in the expression of downstream genes or metabolites to alter a phenotype to desirable states. The applications of genetic engineering are enormous. By genetically engineering plants to contain high levels of cellulose and hemicellulose [1], one may absorb the prohibitive cost of cellulose pretreatment before biomass-to-biofuel conversion. The brain tumor therapy using genetically engineered brain cells has eradicated tumors completely and affects tumor regression [2]. Current *in vivo* genetic engineering is often by trial-and-error, and unavoidably slow and sub-optimal. The few extant *in silico* genetic engineering strategies are seriously hampered by the scarcity of realistic dynamic models of gene regulatory and metabolic networks. However, we anticipate a closing gap between *in vivo* and *in silico* genetic engineering as realistic computational models of networks are made increasingly available by powerful data-driven network reconstruction software from high-throughput systems biology experiments.

Recent work by Deutscher et al. [3] and Nakae et al. [4] provides multiple gene knockout solutions to optimize the concentrations of designated metabolites in static models of metabolic networks. Our work extends to dynamic models, searching the target genes *in silico* from any subset of genes in a gene regulatory network (GRN) for deletion to maximize the concentration of a downstream gene. Using the probabilistic Boolean network model, Faryabi et al. [5] pose an integer programming problem to maximize the benefit of a cancer patient from the treatment which intervenes the activity of a gene over time. The problem is solved using dynamic programming in optimizing a downstream gene by turning on or off only a single target gene. Based on flux balance analysis, Alper et al. [6] and Jin et al. [7] formulate a linear programming problem, to modify the metabolic pathways in wild type *E. coli*. They introduce the method of minimization of metabolic adjustment to revise the objective function to be quadratic for mutants. Both an exhaustive search and a greedy algorithm have been employed to optimize the yield of lycopene synthesis in the metabolic network by overexpressing or deleting three genes. They show that deleting three genes improves the phenotype of interest more effectively than deleting a single gene.

Motivated by the three-gene-deletion advantage, we study the more general multiple gene knockout (GKO) problem. Although we call all variables gene in our terminology, a variable can represent the concentration of a protein, an mRNA, or a metabolite. We use the discrete dynamical system (DDS) model to represent GRNs [8]–[13]. DDS models can be reconstructed from observed trajectories through data-driven methods [14]–[16], some of which can run on parallel supercomputers such as [13]. A nonlinear integer programming problem is formulated to define the GKO problem. We prove the nonlinear integer programming problem to be *NP*-hard. To approach efficiently the global maximum of the nonlinear integer programming problem with a generally non-concave objective function, we transform it to a nonlinear programming problem with fewer decision variables. We offer an algorithm called GKONP to solve the nonlinear programming problem. GKONP prunes insignificant terms in the objective function and takes advantage of the differential evolution algorithm, a parallel global optimization method. We use both the yeast pheromone pathway model and simulated models to demonstrate the performance of the GKONP algorithm.

## Methods

### Mathematical Formulation of the GKO Problem

We introduce the DDS model and formulate a nonlinear integer programming problem to search the optimal regulatory target genes for deletion. Here, we give the problem definition and notations.

#### The DDS Model

We use the DDS model [13] to represent dynamical interactions in GRNs. DDS modeling is data-driven and has been used for characterizing the cell cycle network [9]. The model assumes that the change rate of each gene at the current time point is a linear combination of concentrations of genes at the previous time point. Thus state transitions are independent of each other. Let 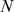 be the number of genes. Let 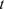 be the discrete time starting from 0. Let 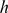 denote the actual time between two consecutive discrete time points. Let 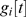 be the concentration of gene 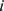 at time 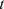. Let 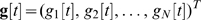 be a state vector of concentrations of all genes at time 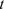. Then, the 1st-order linear DDS model is defined by
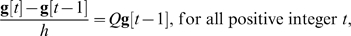
(1)where 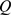 is an 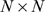 regulation matrix, epitomizing a GRN. 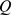 can be estimated with experimental data from wild type under normal and perturbed conditions. Letting 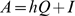, we have
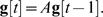
(2)We call 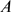 the system matrix. Evidently the solution to the DDS model is
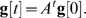
(3)Let 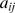 be the entry at row 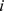 and column 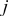 of matrix 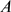. 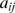 is zero if gene 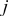 is not a parent (regulator) of gene 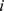. Matrix 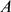 is sparse when the number of parents of each gene is small.

#### Optimal Target Gene Deletion through Nonlinear Integer Programming

Based on the DDS model, a nonlinear integer programming is formulated to maximize a downstream gene by searching regulatory target genes for deletion. We define the binary knockout vector 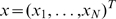. 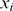 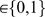 is 1 if gene 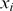 is intact; 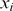 is 0 if gene 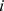 is deleted, equivalent to setting all entries on either row 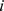 or column 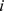 in system matrix 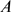 to zero. A GRN with knockout can be represented by a new system matrix
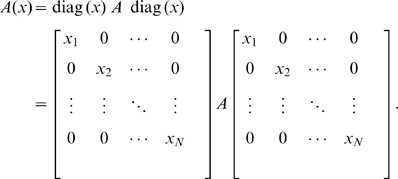
Thus, the knockout DDS solution is 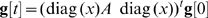. Using the DDS solution, we define the GKO problem to maximize the objective function 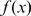, denoting the concentration of gene 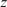 at time 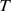, by knocking out a subset of genes:
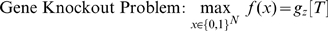
(4)

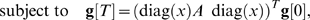
(5)

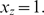
(6)Let 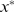 be an optimal solution to the GKO problem. As we want to maximize the concentration of downstream gene 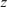, it should not be considered for deletion and hence the constraint 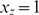.

#### Notations – Path, Weight, and Contribution

**Figure 1:**
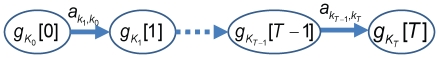
A path over 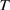 time steps.

We define path, weight of a path, and contribution of a path, to be used in the rest of the paper. A path from gene 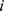 at time 0 to gene 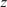 at time 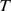 over 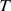 time steps is a 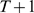 dimensional vector, 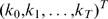, where 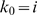 and 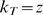. The path is illustrated in Fig. 1.

The weight of a path is 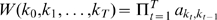. The contribution of gene 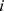 to 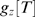 through a path is defined by 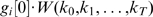. A path is negative/zero/positive if the contribution through the path to 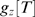 is negative/zero/positive, indicating whether gene 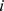 influences 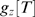 negatively or positively.

### Time Complexity of the GKO Problem

We show that the GKO problem is *NP*-hard by reducing the *NP*-complete vertex cover problem to a special case of the GKO problem. Let 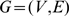 be an undirected graph with a set 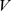 of 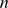 vertices and a set 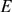 of edges. A vertex cover is a subset of 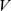 that contains at least one end point of each edge in 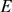. The vertex cover problem is to find a smallest vertex cover of 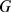. We use 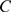 to represent the indices of vertices in a vertex cover of 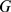.

#### The Vertex Cover Problem Is a Special Case of the GKO Problem

We construct a 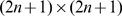 matrix, 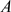, from graph 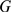 by
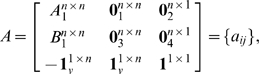
(7)with
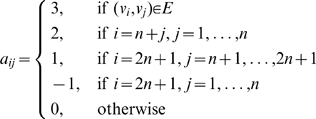
(8)where 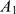 is an 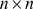 symmetric matrix, the row and column of whose non-zero entry corresponds to an edge in 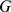, 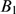 is a diagonal matrix 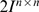, 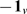 is a 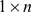 matrix whose entries are all 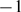, 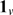 is a 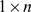 matrix whose entries are all 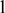, and 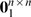, 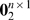, 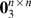, and 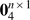 are all zero matrices.

Now, we formulate the GKO' problem of 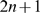 genes, a special case of the GKO problem, as
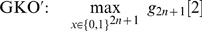
(9)

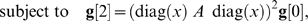
(10)

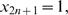
(11)

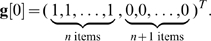
(12)


**Figure 2:**
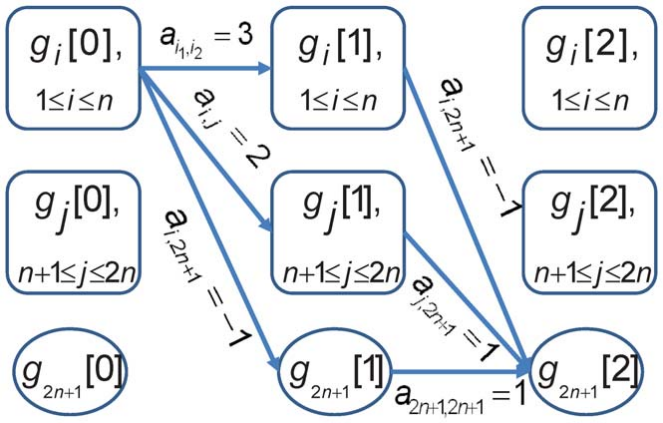
Schematic diagram for the three types of paths influencing 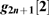.

The 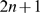 genes in the GKO' problem can be separated into three groups by their indices: 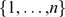, 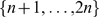, and 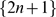. Only paths originating from group one, shown in Fig. 2, influence 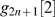. All other paths to 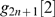, not shown, originating from either group two or three, contribute zero to 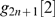. We further define three types of paths, shown in Fig. 2, all originating from some genes in group one, as follows:

- Type 1 path: it goes from a gene in group one at time 0, via another gene in group one at time 1, to gene 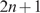 at time 2 with a weight of 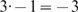. Both gene 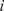 and 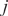 contribute 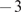 through their corresponding type 1 paths if 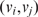 is an edge in graph 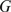 and both gene 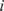 and 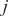 exist in the network. Therefore, the number of type 1 paths is the number of nonzero elements in 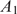.
- Type 2 path: it goes from group one, via group two, to gene 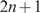 with a weight of 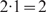. A gene in group one contributes 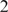 to 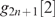 through its corresponding type 2 path if it exists in the network. Therefore, the number of type 2 paths is the number of existing genes in group one.
- Type 3 path: it goes from group one, via gene 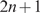, to gene 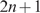 with a weight of 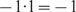. A gene in group one contributes 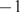 to 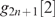 through its corresponding type 3 path if it exists in the network. Therefore, the number of type 3 paths is the number of existing genes in group one.

As the initial state is non-negative, no genes in group two should be knocked out, because doing so would not possibly increase 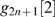, due to type 2 paths being non-negative. Thus, we consider deleting genes from group one as only feasible solutions to the GKO' problem.

Evidently, it takes polynomial time 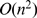 to construct the GKO' problem from the vertex cover problem.

#### The GKO' Problem and the Vertex Cover Problem Are Equivalent

The two problems are equivalent if and only if any smallest vertex cover 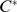 of the vertex cover problem translates to an optimal solution 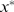 to the GKO' problem and *vice versa*.

Let 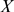 be the set of all feasible solutions to the GKO' problem. Let 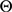 be the power set of 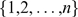 representing all subsets of vertices in 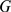. We define a bijective function, 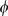, from 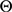 to 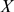 by
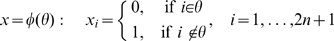
(13)Function 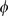 translates any subset 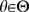 of vertices in 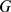 to a feasible solution 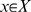 to the GKO' problem with a corresponding objective function value 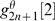. When 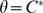, the objective function value is 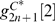.

#### Lemma 1

*If* 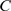 *is a vertex cover of graph* 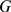, *then* 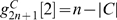.

*Proof.* By Fig. 2, there are three types of paths influencing 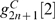. Since 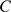 is a vertex cover for graph 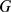, 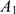 in equation (7) of matrix 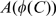 is a zero matrix. That means there is no network between any two genes in group one and, then, genes contribute nothing to 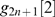 through a type 1 path if we delete all gene 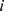 for all 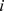 in 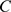 from the GKO' problem. However, each gene 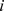 in group one, which is not deleted, contributes two through a type 2 path and negative one through a type 3 path. Therefore,
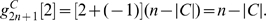



#### Lemma 2

*If* 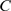 *is a vertex cover of graph* 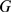, *then* 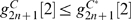.

*Proof.* Since 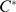 is the index set for a minimum cover, we have
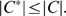



According to Lemma 1, we have.
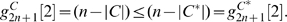



#### Lemma 3

*Let* 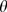 *be a non-vertex-cover subset of vertices. Let* 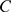 *be a smallest vertex cover that subsumes* 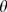. *Then* 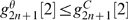 *holds true.*

*Proof.* According to Fig. 2, one additional type 1 path contributes 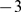 to gene 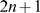 at time two while one additional type 2 path contributes 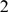 to gene 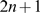 and one additional type 3 path contributes 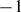 to gene 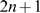.

Since 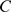 is a vertex cover and 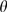 belongs to 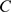, genes in group one have several additional paths to contribute nonzero values to 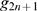 at time two if we only delete gene 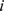 for all 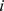 in 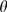 instead of in 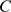. Let the difference of sets 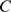 and 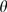 be 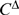. One more gene adding into the network from 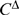 causes more than one additional nonzero element in 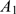 in equation (7). We know that the number of type 1 paths is the number of nonzero elements in 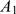. Therefore, the total contribution from the additional type 1 paths is less than
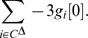
(14)As the number of type 2 or 3 paths is the number of existing genes in group one, the contribution from the additional type 2 paths is
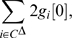
(15)and that from the additional type 3 paths is
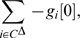
(16)The total contribution of those additional paths is less than
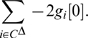
(17)Since the value in equation (17) is negative, value 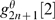 is less than 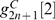 and this lemma is proved.

Combining Lemmas 2 and 3 establishes that 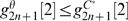 for any subset 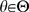 of vertices in 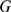 if 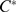 is a smallest vertex cover. Let 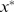 be an optimal solution of GKO' and 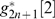 be its maximal value. We have the following two propositions.

#### Proposition 4

*If* 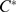 *is a smallest vertex cover of* 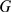, *then* 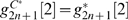.

*Proof.* (By contrapositive) Assume 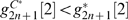. 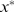 can be translated to 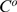 by 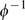. If 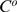 is not a smallest vertex cover, 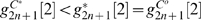 contradicts either Lemma 2 or 3. If 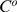 is a smallest vertex cover, we have 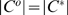. Then 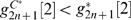 contradicts Lemma 1. Thus, 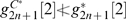. By definition of 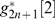, it is also impossible to have 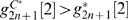. Therefore, we must have 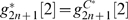.

#### Proposition 5

*Let* 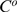 *be* 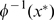. *Then,* 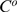 *is a smallest vertex cover of* 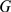.

*Proof.* (By contrapositive) Assume 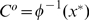 is not a smallest vertex cover of 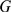. Then one can find a smallest vertex cover 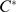 of 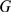. Thus, it must follow by either Lemma 2 or 3 that 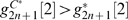, which contradicts the fact that 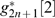 is maximal. Therefore, 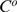 must be a smallest vertex cover with 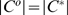.

Propositions 4 and 5 establish that the GKO' and the vertex cover problems are equivalent.

#### The GKO Problem is *NP*-Hard. Theorem 6

*The GKO problem is NP-hard. Proof.* By Propositions 4 and 5, any solution to the vertex cover problem translates to a solution to the GKO' problem and *vice versa*. Since the vertex cover problem is in its most general form, any instance of the vertex cover problem is thus reducible to the GKO' problem. As the vertex cover problem is *NP*-complete and it can be reduced in polynomial time to the GKO' problem, a special case of the GKO problem, the GKO problem is *NP*-hard.

### The Approximation Algorithm of GKONP

As the number of feasible solutions to the GKO problem increases exponentially with network size 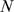, it is impractical to solve it by exhaustive search when 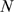 is large. Using the concept of paths, the GKO problem is rewritten to an equivalent nonlinear programming problem. Combining a strategy on pruning the insignificant terms in the objective function and a differential evolution algorithm, we provide a heuristic algorithm to the *NP*-hard GKO problem.

#### Nonlinear Programming for the GKO Problem

We rewrite the nonlinear integer programming problem to an equivalent nonlinear programming problem. Let 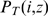 be the collection of paths from gene 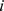 to gene 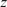 over 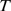 time steps. The sum of contributions of various paths from gene 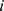 to 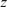 over 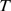 time steps is
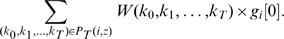
(18)It follows that the objective function 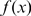 of the GKO problem is the sum of contributions from all genes to gene 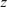:
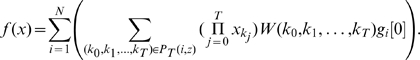
(19)A path 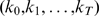 may visit a gene more than once. We extract the unique genes on the path to form a set 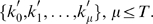 As each element in 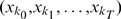 is either zero or one, we have 
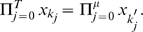
 Then, 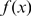 can be rewritten as
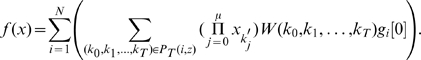
(20)


Only a negative path, 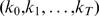, in 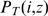 gives a negative term, 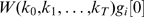, in equation (20). Therefore, we shall delete genes in negative paths to maximize equation (20) and those genes only on non-negative paths need not to be considered for deletion. We denote the collection of genes on negative paths to gene 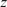 by 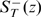. Then, the size of feasible solutions of the nonlinear integer programming problem is scaled down from 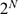 to 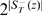.

Let 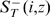 be the collection of genes on negative paths from gene 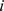 to gene 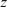. Let 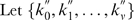 represent the intersection of 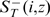 and 
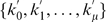
. It follows 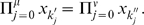 The objective function becomes
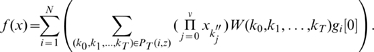
(21)


#### Lemma 7

*If a nonlinear programming problem has objective function* 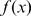 *(* *equation 21* *) and all decision variables* 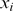, 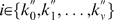, *bounded by* 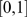, *then there exists an optimal solution which is a vertex of the feasible hypercube.*

*Proof.* Assume 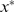, 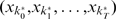, is an optimal solution but not a vertex. Therefore, there must exist an element 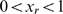 in the solution. Then, the value of objective function (equation 21) with this solution is
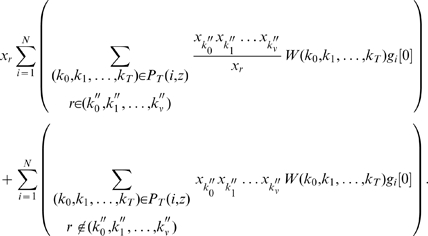
(22)If the value of function (equation 22) is positive at point 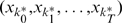, we can increase 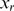 to one to improve the value. Otherwise, we decrease 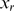 to zero. Since we can improve the value of objective function (equation 21) by moving 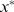 to a vertex of the hypercube search space, this lemma is proved.

By Lemma 7, the original GKO problem becomes Nonlinear Programming for the GKO Problem
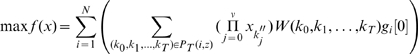
(23)

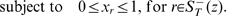
(24)


#### The Filter-Dynamical-Path Algorithm

We introduce the Filter-Dynamical-Path (FDP) algorithm to approximate the objective function 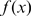 in the form of equation (23). The FDP algorithm, generating the terms of objective function 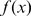 step by step backward from time 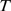 to time 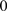, discards insignificant terms at each step. Since the long run behavior of most GRNs shall be stable, 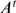 in the DDS model also has to be such when 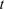 increases. The contributions of most paths will thus vanish and the corresponding terms are removed by the FDP algorithm when time 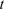 is long enough.

**Figure 3:**
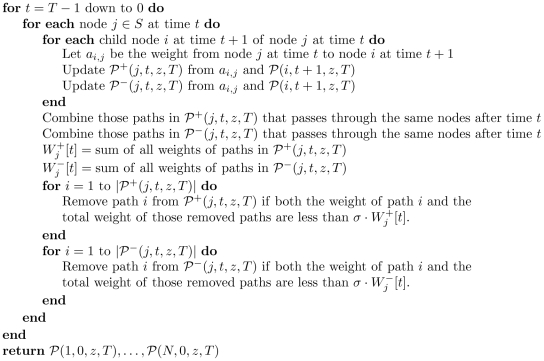
Algorithm 1. Filter-Dynamical-Path(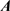, 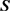, 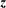, 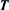, 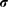).

Let 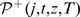 (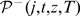) denote the collection of those positive (negative) paths through gene 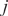 at time 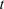 to gene 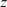 at time 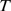 and their weights. 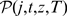 represents the union of 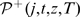 and 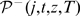. 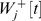 (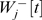) denotes the total weight of positive (negative) paths in 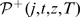 (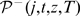). The FDP algorithm, moving backward over time, removes those positive (negative) terms such that the total weight of the related paths is at most 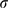 of the total weight of the remaining positive (negative) paths. We call 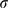 the prune coefficient. Let 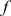 be the approximate value to the true objective function value 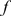. In our simulation study, the relative error is roughly bounded by
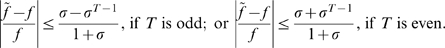
The inequalities suggest that the smaller 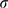 is, the closer the approximation is to the true value. For instance, when 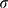 is 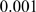 and 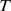 is 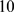, we have 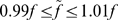. Details of the FDP algorithm is shown as Fig. 3.

#### The GKONP Algorithm

**Figure 4:**
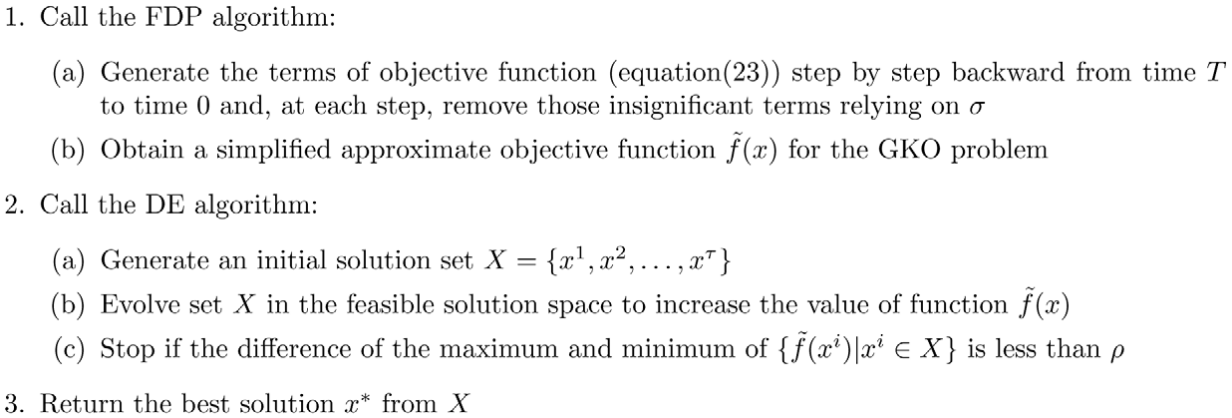
Algorithm 2. GKONP(prune coefficient 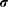, tolerance 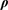).

Based on the nonlinear formulation, we develop a heuristic algorithm to solve the GKO problem. We call it the GKONP algorithm, shown as Fig. 4.

It combines the FDP algorithm and a differential evolution (DE) algorithm for nonlinear programming. The GKONP algorithm simplifies the objective function first by the FDP algorithm and then use the DE algorithm to obtain a final solution to the GKO problem.

The DE algorithm [17], [18] approaches a global maximum of non-concave objective functions as in the GKO problem. The DE algorithm is an evolutionary optimization method. The first step is to generate an initial population of feasible solutions, typically 2 to 50 times of the decision variables. Each individual in the population either remains unchanged or mutates to a new feasible solution in one iteration of evolution. The occurrence of a mutation depends on a trial vector and a probability 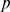. The trial vector combines three other individuals, randomly chosen from the population. If the trial vector is a feasible solution and improves the value of objective function, then the individual mutates to the trial vector with probability 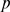. Since the evolution of an individual is independent of others, evolutions of individuals can progress simultaneously and hence can be done in parallel.

## Results

The GKONP algorithm is applied to improve the concentrations of downstream proteins or protein complexes Fus3PP, Fur1PP-Cdc28 (complex N) and Fur1PP-G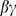 (complex M), involved in the yeast pheromone pathways. Moreover, we evaluate our algorithm on randomly generated DDS models to illustrate its empirical accuracy and running time.

### Optimal Deletion in the Yeast Pheromone Pathways

**Figure 5:**
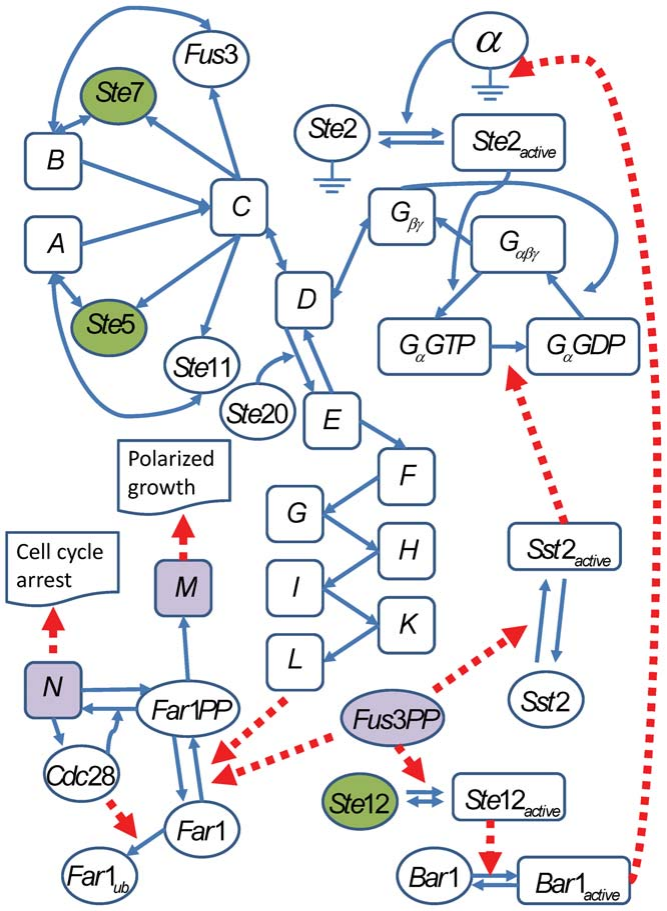
The schematic diagram for the pheromone pathway [14]. The ellipse shapes represent proteins while the rectangle shapes represent protein complexes. The solid lines represent the intracellular reactions while the thick dash lines represent catalysis. We note that the decomposition from complexes E, F, G, H and L to proteins Ste20, 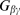, Ste5, Ste11, Ste7 and the dephosphorylation of Fu3PP are not shown in the diagram since they are less dominant than those shown in the pheromone pathway.

We demonstrate our GKONP algorithm using a realistic *Saccharomyces cerevisiae* pheromone pathway model developed by Kofahl et al. [14], shown in Fig. 5. The model is obtained after they studied cell cycle arrest, mating activity, and pheromone sensitivity. The model is publicly available from the BioModels database [19] in the form of a dynamical system model composed of ordinary differential equations (ODEs). The pheromone signaling pathway involves a series of biochemical reactions starting with the receptor of 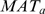 receiving pheromone 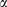 factor from haploid 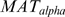. From the cytoplasm, the pheromone signal enters the nucleus to express downstream protein Fus3PP, protein complex N and protein complex M, which together control pheromone sensitivity, cell polarity and cell cycle arrest for preparation of cell fusion between two mating haploid yeast cells, 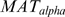 and 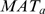. Haploid 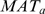 cannot stop cell cycle to mate with 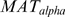 if the concentrations of the three protein products are low. Therefore, it is desirable to engineer the yeast to improve these downstream protein products to increase mating activity.

**Figure 6:**
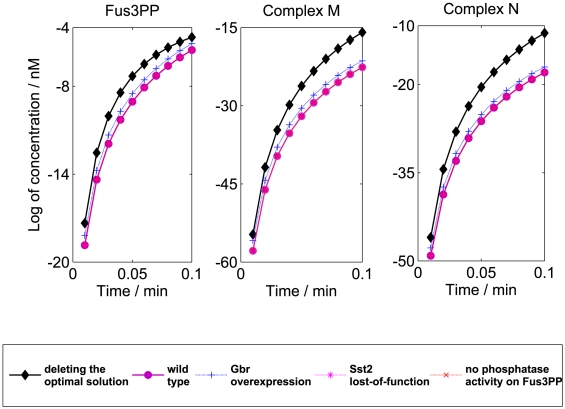
The optimal solution from the GKONP algorithm vs. the wild type and three observed mutants. The GKONP optimization (top diamond lines) returned higher concentrations of desired downstream proteins (Left: Fus3PP, Middle: Complex M, and Right: Complex N) than both the wild type and the *in vivo* trial-and-error mutants [14]. Left: the optimal target genes are Ste5, Ste7 and Ste12. Middle and right: the optimal target gene are both Ste12. The dynamics of wild type, the mutant (Sst2 lost-of-function), and the mutant without phosphatase activity on Fus3PP overlap in all three plots.

Thus, we applied the GKONP algorithm to identify upstream knockout genes to improve the concentrations of the three downstream protein products. By simulation using the ODE model, we first generated continuous-time trajectories. Second, we sampled them every 0.6 seconds from 0 to 6 seconds to obtain discrete-time trajectories. Then we reconstructed a DDS model (Appendix S1) from the discrete-time trajectories using a data-driven method [13] that balances goodness-of-fit and model complexity. The DDS model captures the transient dynamics in the pathway in which the three protein products are actively expressed. Using the DDS model as input, we ran the GKONP algorithm three times to search for three optimal target gene sets in the pathway for improving the concentrations of downstream products of Fus3PP, complex N and complex M, respectively. A feasible solution is any subset of {Ste2, Ste5, Ste11, Ste7, Ste20, Ste12, Fus3PP, Bar1, Far1PP, Cdc28}. The optimal target gene sets for improving each of Fus3PP, complex N and complex M are {Ste5, Ste7, Ste12},{Ste12} and {Ste12}, respectively. The optimal target genes obtained through GKONP algorithm were validated in the original ODE model. By assigning zero values to the deleted genes, we simulated the modified dynamics of the engineered ODE model. Figure 6 presents the transient dynamics, computed from the original ODE model as a validation, of the concentrations of Fus3PP, complex N and complex M in the wild type and five mutants from 0 to 6 seconds. The modified dynamics are compared with those of wild type and three observed mutants which have high concentrations of at least one of those three downstream protein products. These three observed mutants include a mutant whose 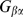 is overexpressed (double amount of 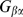) [20], a mutant whose Ste2 loses function (the hydrolysis of 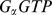 to 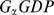 is almost stopped) [21] and a mutant that has no phosphatase activity on Fus3PP (the concentration of Fus3PP is strongly increased for a long time) [22]. From this figure, we demonstrate that, by deleting optimal subsets of target genes, the concentrations of all three desirable downstream protein products are higher than the wild type and the trial-and-error *in vivo* mutants.

### Accuracy and Time Efficiency

Nine randomly generated DDS models were used to test the performance of the GKONP algorithm. The model sizes are 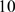, 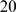 and 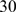, with 3 instances for each size. As each gene is only regulated directly by a very small portion of its network, without loss of generality, we constrained each DDS model such that each gene has less than three parent genes. Thus, no more than three entries were non-zero in each row of matrix 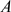, whose values were between 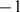 to 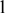. Each gene had one unit of concentration at time 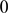. We applied the GKONP algorithm on the DDS models to maximize the concentration of the first gene at time 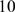. Prune coefficient 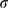 was set to 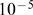. We also ran the brute force exhaustive search algorithm on the DDS models as a reference for performance evaluation of the GKONP.

Table 1 shows the GKONP algorithm approaches optimal solutions accurately because all six approximations for the 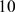 gene and 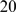 gene DDS models are identical to the optimal values, given by the exhaustive algorithm.

**Table 1:** Approximate GKONP solutions versus the optimal solutions.

Data set	# Genes	Optimal value	GKONP value
1	10	0.1734801	0.1734801
2	10	0.3546365	0.3546365
3	10	0.01020017	0.01020017
4	20	0	0
5	20	0.02599726	0.02599726
6	20	0.1683985	0.1683985

**Figure 7:**
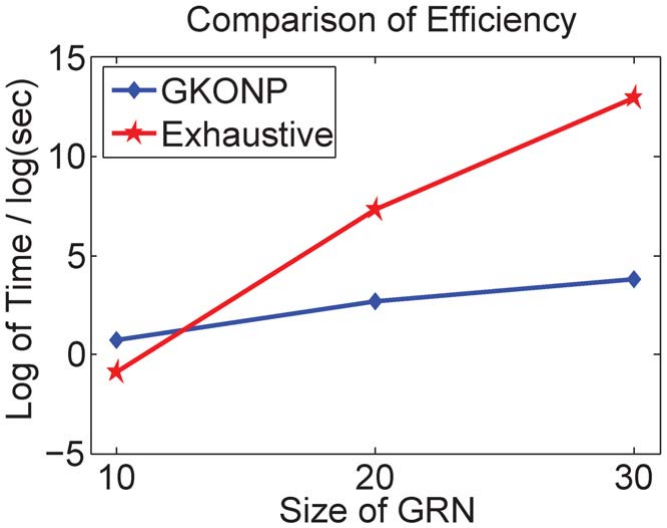
Average running time of the GKONP algorithm and the exhaustive search algorithm. The GKONP algorithm is represented by the blue line with diamonds while the exhaustive search algorithm is by red line with circles.

The running time as a function of model sizes is shown in Fig. 7. The exhaustive search algorithm for the 30-gene DDS models took more than five days and we rounded the time to five days. The speedup of the GKONP algorithm ranges from 0.09 to 1.42 in the 10-gene models, 47 to 11,388 in the 20-gene models, and 4,763 to 165,390 in the 30-gene models. Therefore, Figure 7 suggests that the GKONP algorithm is much more efficient than the exhaustive search algorithm.

**Figure 8:**
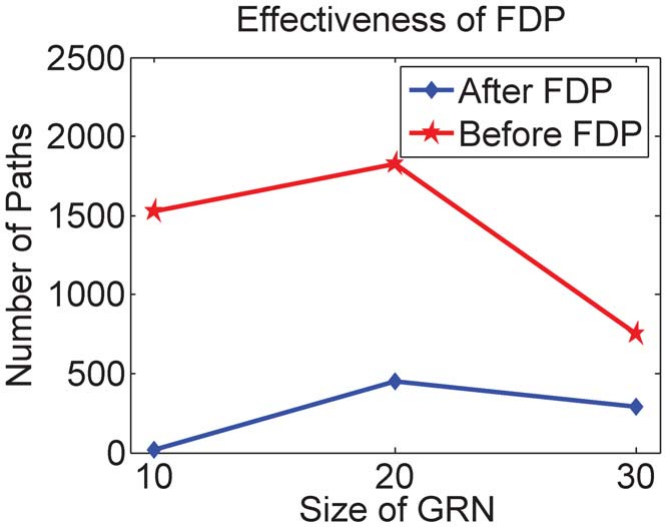
Average path reduction by the FDP algorithm. The red line with circles represents the number of paths before FDP reduction while the blue line with diamonds represents the number after FDP reduction.

In Fig. 8 the number of paths decreases significantly after the FDP algorithm is applied, so that the DE algorithm runs in a reduced search space.

Since the GKONP searches negative paths of a DDS model, it was slower with 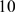 genes than the exhaustive search. However, as the number of genes increases to 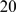 and 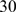, our approach has extraordinary speedup over the exhaustive search. With the same model size, the running time advantage of GKONP becomes evident when the topology of a DDS model contains either few negative paths or very few genes in negative paths. For instance, a 20-gene DDS model had 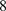 genes in negative paths and the GKONP yielded a speedup of 11,388 versus another 20-gene model with a speedup of only 47.

This simulation study demonstrates empirically that the GKONP algorithm has achieved good accuracy in a practical amount of running time.

## Discussion

We have established that the optimal *in silico* target gene deletion problem is challenging, by showing that a nonlinear integer programming formulation of the GKO problem based on the DDS model is *NP*-hard. A nonlinear programming solution is provided that combines heuristics based on the sparsity of typical GRNs and a parallel differential evolution algorithm for nonlinear programming. Multiple simultaneous gene deletion is handled in our approach, while all existing strategies delete one gene at a time. Our algorithm GKONP has shown its substantially reduced running time and comparable accuracy with the optimal solutions using exhaustive search algorithms. Demonstration of our solution on a realistic model of yeast pheromone pathways has suggested potential impact of our work. Hopefully, ideas presented in this paper will bring out potentially harder but biologically more viable computational problems for richer formulation of the target gene deletion problem, based on more complex dynamical system models of gene regulatory and metabolic networks with additional constraints on side effects.

## Supporting Information

Appendix S1 The DDS Model for the Pheromone Pathway
(0.03 MB PDF)
