# Supplementary material for: Optimal In Silico Target Gene Deletion through Nonlinear Programming for Genetic Engineering
Source: PLoS One. 2010 Feb 24;5(2):e9331. doi: 10.1371/journal.pone.0009331 (PMC2827548; doi:10.1371/journal.pone.0009331)
Supplement: Appendix S1 — The DDS Model for the Pheromone Pathway (0.03 MB PDF) [file pone.0009331.s001.pdf]

The DDS Model for the Pheromone Pathway

$$
\begin{aligned}
\alpha[t{+}1] &= \alpha[t] \\
Ste2[t{+}1] &= 12.14\alpha[t]{+}0.27Ste2[t]{-}0.88Ste2a[t] \\
Ste2a[t{+}1] &= 0.23\alpha[t]{-}0.01Ste2[t]{+}0.97Ste2a[t] \\
G_{\alpha\beta\gamma}[t{+}1] &= -0.04Ste2a[t]{+}G_{\alpha\beta\gamma}[t]{-}0.1G_{\beta\gamma}[t]{-}44.26G_{\alpha}GDP[t] \\
G_{\alpha}GTP[t{+}1] &= 0.06Ste2a[t]{+}G_{\alpha}GTP[t]{-}3061.34Sst2[t] \\
G_{\beta\gamma}[t{+}1] &= 0.02Ste2a[t]{-}7.1G_{\beta\gamma}[t]{+}182.62G_{\alpha}GDP[t]{+}0.01C[t]{+}1787.34D[t]{-}0.98E[t]{-}37.53F[t] \\
&\quad {+}312.41G[t]{-}1.75H[t] \\
G_{\alpha}GDP[t{+}1] &= 1.07G_{\alpha}GDP[t] \\
C[t{+}1] &= 4.4G_{\beta\gamma}[t]{+}0.55C[t]{-}966.78D[t]{+}0.54A[t]{+}0.68B[t] \\
D[t{+}1] &= 0.1G_{\beta\gamma}[t]{-}19.47D[t] \\
Ste5[t{+}1] &= 0.37C[t]{+}0.51Ste5[t]{-}0.12A[t]{-}0.66E[t]{+}61.61F[t]{-}554.98G[t]{+}37.96H[t]{-}668.24L[t] \\
Ste11[t{+}1] &= 0.37C[t]{-}0.49Ste5[t]{+}Ste11[t]{-}0.12A[t]{-}0.66E[t]{+}61.61F[t]{-}554.98G[t]{+}37.96H[t]{-}668.24L[t] \\
A[t{+}1] &= 4.99Ste5[t]{-}2.5A[t]{-}5.39B[t] \\
Ste7[t{+}1] &= 0.11C[t]{+}0.27Ste7[t]{+}0.02B[t]{-}2.87E[t]{+}149.47F[t]{-}1079.63G[t]{+}17.5H[t]{-}146.76L[t] \\
Fus3[t{+}1] &= 0.97C[t]{+}1.55Ste7[t]{+}0.55Fus3[t]{+}0.32B[t]{-}30.29E[t]{+}1840.63F[t]{-}14175.33G[t]{+}481.28H[t] \\
&\quad {-}38293.62L[t]{+}13448.74Fus3PP[t] \\
B[t{+}1] &= -19.86A[t]{+}9.74Ste7[t]{+}2.74Fus3[t]{-}0.68B[t] \\
Ste20[t{+}1] &= -286.53D[t]{+}Ste20[t]{+}21.06E[t]{-}742.39F[t]{+}4754.87G[t]{-}45.01L[t] \\
E[t{+}1] &= 57.82D[t]{+}0.78E[t] \\
F[t{+}1] &= 0.13E[t]{+}0.23F[t] \\
G[t{+}1] &= 0.63F[t]{-}3.8G[t] \\
H[t{+}1] &= 4.94G[t]{+}0.09H[t] \\
I[t{+}1] &= 1.9H[t]{-}12.08I[t]{+}23.57K[t] \\
L[t{+}1] &= 9.01I[t]{-}459.42L[t]{+}0.81K[t] \\
Fus3PP[t{+}1] &= 3.2I[t]{-}83.68Fus3PP[t]{+}0.24Ste12a[t] \\
K[t{+}1] &= 110.06L[t]{-}3.25K[t] \\
Ste12[t{+}1] &= -51.07Fus3PP[t]{+}Ste12[t]{+}0.21Ste12a[t] \\
Ste12a[t{+}1] &= 64.14Fus3PP[t]{+}0.5Ste12a[t] \\
Bar1[t{+}1] &= -0.69Ste12a[t]{+}Bar1[t]{+}1.64Bar1a[t] \\
Bar1a[t{+}1] &= 0.41Ste12a[t]{+}0.47Bar1a[t] \\
Bar1aex[t{+}1] &= Bar1aex[t] \\
Far1[t{+}1] &= -62.52Fus3PP[t]{+}0.94Far1[t]{+}5225926.11Far1PP[t] \\
Far1PP[t{+}1] &= 8.59Far1PP[t]{+}1486.11M[t]{-}36.55N[t] \\
Far1U[t{+}1] &= 0.06Far1[t]{+}Far1U[t] \\
M[t{+}1] &= 1.69M[t] \\
N[t{+}1] &= 0.98Far1PP[t]{-}0.86N[t] \\
Cdc28[t{+}1] &= Cdc28[t] \\
Sst2[t{+}1] &= 2.68Sst2[t]
\end{aligned}
$$
